# Intracellular kinetics of the androgen receptor shown by multimodal Image Correlation Spectroscopy (mICS)

**DOI:** 10.1038/srep22435

**Published:** 2016-03-03

**Authors:** Chi-Li Chiu, Katherin Patsch, Francesco Cutrale, Anjana Soundararajan, David B. Agus, Scott E. Fraser, Daniel Ruderman

**Affiliations:** 1 Center for Applied Molecular Medicine, University of Southern, California, USA; 2 Translational Imaging Center, University of Southern, California, USA

## Abstract

The androgen receptor (AR) pathway plays a central role in prostate cancer (PCa) growth and progression and is a validated therapeutic target. In response to ligand binding AR translocates to the nucleus, though the molecular mechanism is not well understood. We therefore developed multimodal Image Correlation Spectroscopy (mICS) to measure anisotropic molecular motion across a live cell. We applied mICS to AR translocation dynamics to reveal its multimodal motion. By integrating fluorescence imaging methods we observed evidence for diffusion, confined movement, and binding of AR within both the cytoplasm and nucleus of PCa cells. Our findings suggest that in presence of cytoplasmic diffusion, the probability of AR crossing the nuclear membrane is an important factor in determining the AR distribution between cytoplasm and the nucleus, independent of functional microtubule transport. These findings may have implications for the future design of novel therapeutics targeting the AR pathway in PCa.

The androgen receptor (AR) regulates genes involved in the development and maintenance of the male phenotype, and also plays a role in the growth and survival of prostate cancer (PCa). AR is a ligand-activated transcription factor belonging to the class I subgroup of the nuclear receptor superfamily^1^. In the absence of hormone ligand (testosterone and dihydrotestosterone), unstimulated AR is located preferentially in the cytoplasm. There, AR complexes with heat shock proteins and various co-chaperones, which maintains AR in a conformation capable of ligand binding and protects the receptor from proteolysis^2^. Upon activation by agonist ligand binding, the predominantly cytoplasmic AR translocates into the nucleus, where the AR-ligand complex binds androgen response elements in gene promoter and enhancer regions of multiple target genes^3^,^4^. Subsequently, the AR complex initiates the recruitment of specific transcriptional co-regulators, which alter local chromatin structure in order to enhance transcription initiation, and induces transcription of target genes^5^.

The interactions of AR with DNA and protein, as well as molecular crowding and intracellular structural barriers, can give rise to complex kinetic behavior of AR in a ligand-dependent manner. Alteration of AR nuclear/cytoplasmic localization and of its interactions with other molecules are tightly associated with PCa growth, progression, and resistance to therapy^6^. Taxane microtubule-targeting agents, including docetaxel and paclitaxel, have enjoyed success in the treatment of metastatic PCa and are the first-line treatment choice for the castration-resistant metastatic PCa. Taxanes have been demonstrated to bind to microtubules and prevent their disassembly, thereby suppressing microtubule dynamics and leading to mitotic arrest and apoptosis^7^. This was believed to be the sole mechanism of taxane action in PCa until recently, when studies demonstrated that taxanes may also act through a mechanism involving inhibition of AR nuclear translocation^8^,^9^. This suggests that a direct inhibitory effect on AR also contributes to taxane efficacy^10^. This notion is supported by several recent clinical observations of cross-resistance in castration-resistant prostate cancer between hormonal therapy and taxane-based chemotherapy, suggesting a common culprit may underlie this phenotype^11^,^12^,^13^,^14^,^15^. Combined with reports showing that dynein mediates AR trafficking, the profound cytoplasmic sequestration of AR following taxane treatment implicates microtubules’ role in the shuttling of AR from the cytoplasm to the nucleus^8^. However, evidence of this mechanism in live cells is still lacking. Revealing the details of AR’s kinetics may help guide the development of PCa treatment strategies.

Recently developed microscopy techniques have greatly improved our understanding of nuclear receptor kinetics, including AR^4^,^5^,^16^,^17^,^18^,^19^,^20^. A common approach to probing molecular dynamics is through fluorescence recovery after photo-bleaching (FRAP)^18^,^19^,^20^, which measures a fluorescent particle’s diffusion coefficient by selectively bleaching a cellular region and observing the return of fluorescence to that region. However, due to the large number of experimental variables, the variety of analytical approaches and the inaccuracy of FRAP at short time intervals, the results of these quantifications have failed to provide a consistent view on transcription factor mobility and the nature and timing of their interactions with DNA^21^. Other groups have implemented the fluorescence correlation spectroscopy (FCS) approach^4^,^5^, a method that can assess both diffusion and binding. However, FCS only measures fluctuations at a single location. Furthermore, most studies have focused on the AR kinetics in the nucleus. The dynamical picture of AR across different cellular compartments is still lacking, and, to the best of our knowledge, the potential presence of anisotropic AR motion has not been discussed.

Imaging correlation spectroscopy (ICS) is an attractive approach to probe intracellular dynamics at wide spatiotemporal scales. It works well with genetically encoded fluorescent proteins, and requires minimum perturbation of the biological system. We specifically applied raster image correlation spectroscopy (RICS)^22^,^23^ to capture the fast diffusion of AR. By exploiting the pixel acquisition time structure of raster image scanning from confocal imaging, molecular dynamics on time scales ranging from microseconds to milliseconds can be measured by RICS. In comparison with RICS, FRAP has limited ability to map diffusion coefficients, as only a few, preselected regions can be measured.

While RICS provides isotropic diffusion information, to further investigate the anisotropic as well as slower component of AR dynamics, we needed an extra dimension. For this purpose, we developed a novel technique that we named multimodal Image Correlation Spectroscopy (mICS) to extract the isotropic as well as anisotropic AR motion from image stacks by fitting correlations to a two-component function. mICS analysis is based on spatiotemporal image correlation spectroscopy (STICS)^24^ to capture slower, potentially anisotropic motion. Since its introduction, the STICS approach has been used mainly with frame rates on the one second time scale for protein directional movement^25^,^26^,^27^. An extension of STICS, including iMSD and related techniques^28^,^29^,^30^,^31^,^32^, was recently developed to extract the protein diffusion law by fitting the series of correlation functions, in the form of a mean-square displacement versus time-delay plot. Correlation of the time trace at different lag times results in a decay curve that could be fitted with a model function to extract information about molecular dynamics. We developed mICS based on the idea of iMSD and generalized it to include an anisotropic component.

In the present study, we quantitatively measured AR kinetics across the cell and the changes due to agonist ligand binding by combining RICS and mICS analysis. We observed diffusion, confined movement, and binding of AR within both cytoplasm and nucleus. Combining our experimental data with simulations, our findings suggest that with the presence of diffusion in the cytoplasm, the probability of AR crossing the nuclear envelope is a key factor in determining AR translocation, independent of the presence of functional direct transport in the cytoplasm. This suggests that taxane-induced AR translocation blockade may involve mechanisms other than arresting dynein-mediated AR transport.

## Results

### Diffusion contributes to GFP-AR distribution in a cell

**Figure 1:**
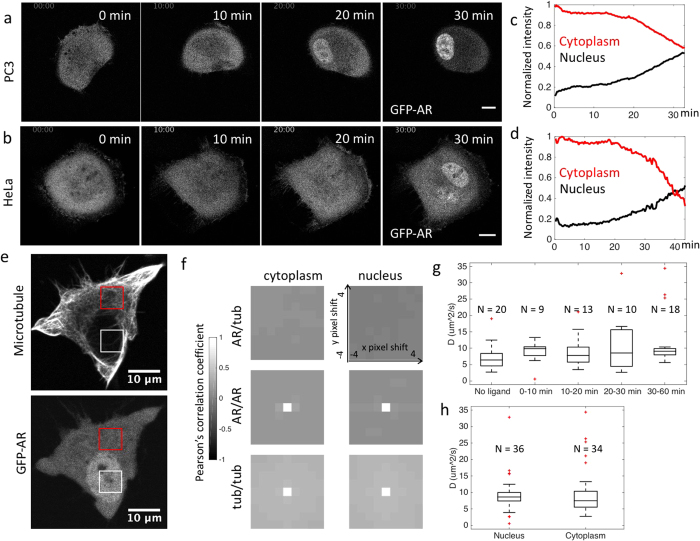
Diffusion contributes to GFP-AR distribution in a cell. (**a**) Time lapse images of a PC3 cell expressing GFP-AR treated with Cl-4AS-1 at 0 minutes. GFP-AR shows clear translocation from the cytoplasm to the nucleus around 20 minutes. Notice the distribution of GFP-AR in the cytoplasm is relatively uniform even with the presence of agonists. (**b**) Time lapse images of a HeLa cell expressing GFP-AR treated with Cl-4AS-1 at 0 minutes. GFP-AR translocation dynamics is similar to (**a**). Scale bar: 10 μm. (**c**,**d**) GFP-AR cytoplasm (red line) and nucleus (black line) normalized total intensity change over time from time lapse of (**a**,**b**). Images were acquired at 15 s/frame and 20 s/frame, respectively. (**e**) A PC3 cell expressing GFP-AR stained with SiR-tubulin that highlights microtubules, stimulated with Cl-4AS-1 for 24 minutes. While SiR-tubulin shows fibrous structure, GFP-AR is uniformly distributed in the cytoplasm. (**f**) Unlike the auto-correlations which represent 100% co-localization at 0 pixel shift (Pearson’s correlation coefficient (r_p_ = 1), the cross-correlation of GFP-AR and SiR-tubulin showed no strong correlation for all pixel shifts between -4 and 4, indicating no clear co-localiztion of SiR-tubulin and GFP-AR. (**g**) GFP-AR diffusion coefficient measured before and after ligand (Cl-4AS-1) treatment. No significant difference was detected among these groups, suggesting the mobility of the diffusive GFP-AR was not significantly affected by the addition of AR agonists (1 way ANOVA, p value = 0.58). (**h**) GFP-AR diffusion coefficient measured by RICS at nucleus (D = 9.25 ± 5.27 μm^2^/s) and cytoplasm (D = 9.72 ± 7.3 μm^2^/s). No significant difference was found between GFP-AR diffusion rates in cytoplasm and nucleus (two-sided t test, p value = 0.76).

We used a clonal PC3 cell line stably expressing GFP-AR as our model system to study AR kinetic changes due to the presence of AR agonists. PC3 is a metastatic human prostate cancer cell line with low AR expression, and was favored in this study to limit interference from non-fluorescent endogenous AR. To verify the functionality of our model system, we analyzed AR expression levels and AR-dependent gene transcription in our cell line. Western blot quantification revealed a 2-fold overexpression of AR in our PC3 GFP-AR cell line compared to endogenous AR within the LNCaP cell line (see Supplementary Fig. S1a), suggesting our model system did not contain unphysiological levels of AR. Luciferase assays confirmed ligand stimulated activation of genes containing androgen response elements, demonstrating GFP-AR’s physiological functionality (see Supplementary Fig. S1b). For ligand stimulation, we chose the synthetic AR agonist Cl-4AS-1 due to its high photo-stability. Many commonly used AR agonists such as R1881 are photodegradable, and thus not suitable for time-lapse fluorescent imaging. We established the Cl-4AS-1 induced AR translocation time scale by time-lapse imaging and quantifying the AR intensity change in the cytoplasm and the nucleus. The same measurement was performed with HeLa cells transiently expressing GFP-AR. The similar pattern of intensity change between PC3 and HeLa cells showed that the GFP-AR translocation dynamics is comparable in different cell lines (Fig. 1a–d). In both PC3 and HeLa cell lines, translocation can be seen in 20 to 30 minutes, similar to previous reports^3^,^33^.

AR and microtubules were previously shown through fixed cell immune-staining to have partial co-localization^8^. To see whether AR’s spatial distribution is correlated with microtubule locations in live cells, we stained live PC3 GFP-AR cells with SiR-tubulin to visualize endogenous microtubules. GFP-AR and SiR-tubulin showed distinct patterns (Fig. 1e): the former was homogeneously distributed throughout the cell, likely due to diffusion, whereas the later was, as expected, localized in fibers. We estimated the degree of co-localization by calculating the spatial cross-correlation^34^ of GFP-AR and SiR-tubulin in the cytoplasm and nucleus (Fig. 1e, red box and white box, respectively). The cross-correlation of GFP-AR and SiR-tubulin showed no strong correlation for all pixel shifts between −4 and 4, with 42 nm/pixel (r_p_ < 0.02 for nucleus and r_p_ < 0.14 for cytoplasm, with no identifiable correlation peak). We conclude that the majority of GFP-AR was not bound to microtubules and was likely free to diffuse throughout the cytoplasm. The relatively uniform distribution of AR persisted over the course of agonist-stimulated AR translocation, as shown in Fig. 1a,b, further suggests the constant existence of AR diffusion.

To measure the diffusion coefficient of GFP-AR, we applied RICS within the cytoplasm and nucleus at different stages during AR translocation. AR diffusion coefficients showed no significant changes during the course of Cl-4AS-1 treatment (Fig. 1g, one way ANOVA, p value = 0.58, N: number of cells); the mean diffusion coefficient ranged from 7.2 to 11.8 μm^2^/s. There was also no significant difference in AR diffusion rates in the cytoplasm or nucleus (Fig. 1h, two-sided student’s t test, p value = 0.76). Overall, the AR diffusion coefficient measured using RICS was 9.2 ± 6.2 μm^2^/s, and diffusive AR existed at all stages of translocation, in both cytoplasm and nucleus. A few measurements showed much higher diffusion coefficients (outliers in Fig. 1g,h), potentially due to different surrounding environment at sub-cellular locations. As a control, we measured eGFP diffusion in PC3 cells using the identical experimental set up. The cytoplasmic eGFP diffusion coefficient was 30.8 ± 6.3 μm^2^/s, in line with the previously reported GFP diffusion coefficient in eukaryotic cytoplasm (27 μm^2^/s in CHO cells)^35^. Our results suggest that GFP-AR diffusion is about three times slower than the fluorescent protein alone. If AR’s diffusion obeys the Stokes-Einstein equation in the cytoplasm^36^, the addition of GFP molecule (27 kDa) to AR (110 kDa) will only result in 6% change in diffusion rate compared to endogenous AR. This small difference is unlikely to affect our conclusions.

### mICS analysis-method overview

**Figure 2:**
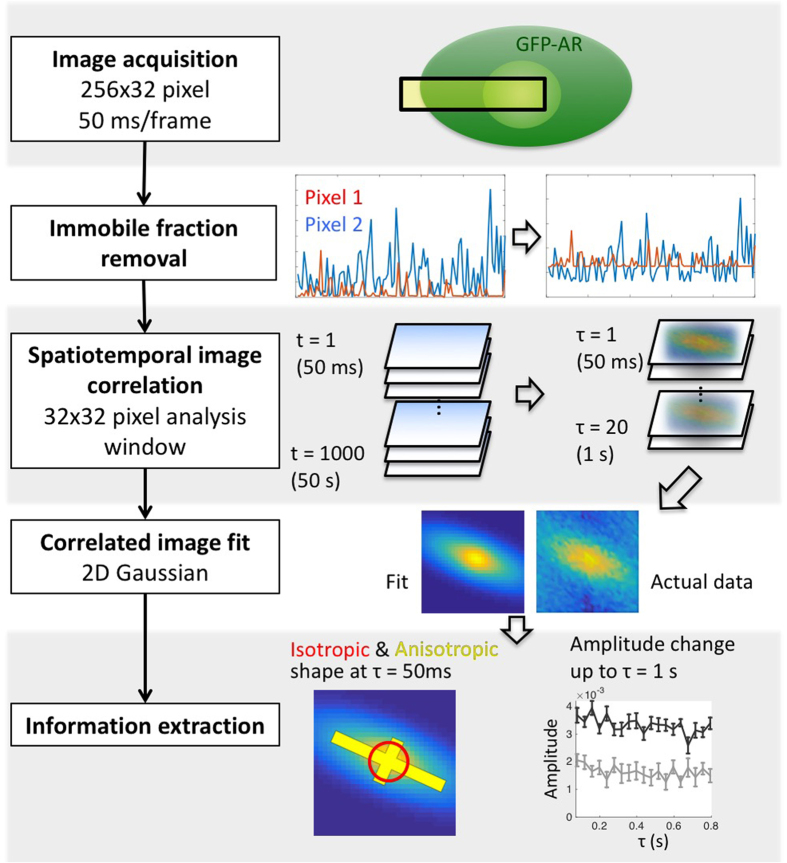
Workflow of mICS analysis for kinetic measurement. To analyze the GFP-AR anisotropic motion as well as its slower kinetics, we implemented mICS analysis. *Image acquisition:* First, images were acquired at 256 × 32 pixel format to capture both the cytoplasm and the nucleus portions, while maintaining the acquisition speed close to 50 ms/frame by a conventional confocal microscope. 1000 frames were acquired for each measurement. *Immobile fraction removal:* The acquired time stack was corrected for photo-bleaching and removing immobile fractions so that the correlation is only consists of pixel intensity fluctuation. *Spatial temporal image correlation:* mICS was applied in 32 × 32 pixel windows, transforming the time series (t = 1 to 1000) into a time delay series (τ = 1 to 20, corresponds to time delay = 50 ms to 1 s) of pixel-shift correlation. *Correlated image fit:* The time delay series was then fitted with two 2D Gaussians to extract the isotropic diffusion/binding as well as the directional confined particle movement. *Information extraction:* The fitting result was presented both schematically to illustrate its size and anisotropic directionality, and in detail to show the change over τ. For detailed descriptions see *Method: mICS analysis*.

While RICS provides isotropic diffusion information, to further investigate the anisotropic as well as the slower component of AR dynamics, we developed mICS analysis. An overview of this method is depicted in Fig. 2. The main differences between the analysis described in this study and the original STICS and iMSD approaches lie in the fitting step and data interpretation. To more accurately describe the complex nature of AR dynamics that is composed of isotropic motion (diffusion and binding) as well as anisotropic motion (confined movement and potential direct transport), we implemented two-component Gaussian fitting that extracts these two features explicitly. Furthermore, we preserved spatial dynamic information and the heterogeneity within a cell. Multiple levels of dynamical aspects were elucidated through this analysis: The shape arises from STICS that indicates the orientation and extent of anisotropic motion; the relative abundance of isotropic and anisotropic population; the time delay correlation plot that gives the information of diffusion/binding rates.

With mICS, the frame rate acts as a temporal filter that discriminates the fast diffusive component from the slower motion. We simulated particles with varying diffusion coefficients to show the dynamic range that can be captured by our measurement parameters (see Supplementary Fig. S2a). At 50 ms/frame, the fast diffusion (D > 1 μm^2^/s) resulted in weak correlation, whereas slower diffusion gave rise to the isotropic component in the correlation image, and the amplitude of this isotropic component decreased with longer delay time. We further demonstrated the correlation behavior with free GFP diffusion in the cell, fluorescent beads diffusing in glycerol solution, and Hoechst dye that strongly bound to DNA in the nucleus (see Supplementary Fig. S2b). To understand the possible origins of anisotropic correlation, we also simulated different anisotropic motions (see Supplementary Fig. S3), and showed that the anisotropic component can arise from oscillating boundaries or oscillating structure with anisotropic shape. The mere existence of a boundary (e.g., nuclear envelope) will not contribute to the anisotropic component.

### GFP-AR kinetics across the cell shown by mICS analysis

**Figure 3:**
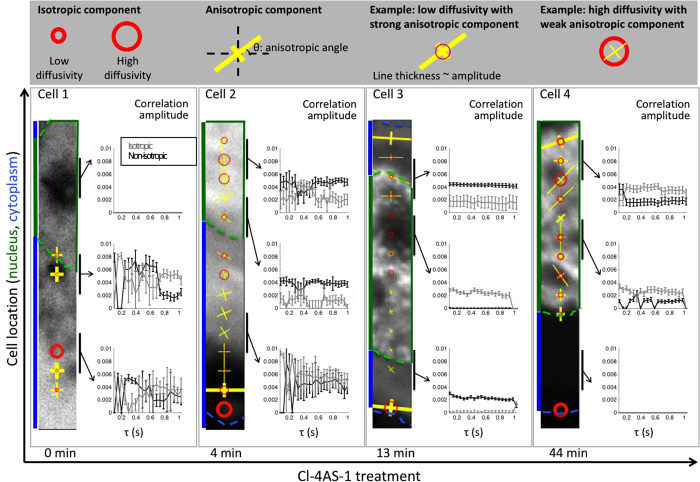
GFP-AR kinetics across cell shown by mICS analysis. We applied mICS analysis to images of GFP-AR expressing PC3 cells under Cl-4AS-1 treatment. A 32 × 32 pixel analysis window at a step size of 16 pixels (half analysis window overlap) was applied along the 256 × 32 pixels image stack. At each analysis window, we overlaid with the kinetic features at τ = 1 to the original images by the following symbol: The isotropic diffusion/binding is represented as red circle, where the diameter corresponds to the FWHM from the isotropic Gaussian fit; the anisotropic confined movement is represented as cross, where the FWHM of the Gaussian long and short axis correspond to the length of the yellow lines. The circle/line thickness is based on the relative amplitudes of those components. The time delay series of selected locations were depicted to show the kinetic timescale. The error bar was calculated based on the Hessian matrix. Within the nucleus, GFP-AR showed striking transition from no detectable slow motion before Cl-4AS-1 treatment, to long, consistent binding. Within the cytoplasm, GFP-AR showed more heterogeneity. Note that the anisotropic component showed consistent amplitude over τ, indicating the confinement is stable at second time scale.

Applying mICS to GFP-AR dynamics, we not only saw isotropic binding, but also identified anisotropic components in multiple cellular locations (Fig. 3). Here we used the size of the circle to represent the degree of diffusion, and the cross to represent the principal axes of the anisotropic component. Within the nucleus, GFP-AR showed a striking transition from no detectable correlation before Cl-4AS-1 treatment, suggesting the majority of GFP-AR to be diffusive, to long, consistent correlation after adding ligand. This AR agonist-induced binding in the nucleus has been reported previously in other studies^19^,^20^,^37^. Within the cytoplasm, GFP-AR showed the decreased diffusion after Cl-4AS-1 treatment. There was more heterogeneity within the cytoplasm, where we found both isotropic and anisotropic motion. Note that the anisotropic component showed consistent amplitude over the delay time, indicating a stable confinement at the second time scale. However, we did not detect directional movement (such as microtubule transport), which would be indicated by a shift in the Gaussian’s center location.

**Figure 4:**
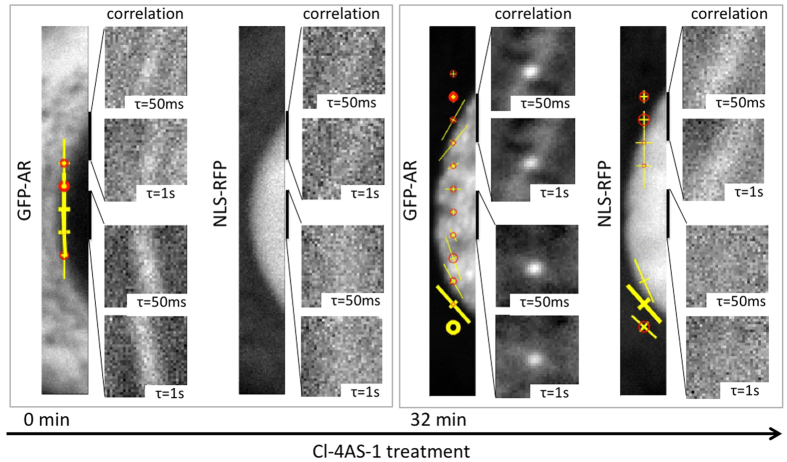
Anisotropic GFP-AR movement results from structural confinement and slow kinetics. Before adding Cl-4AS-1, there was no significant directionality of GFP-AR along the nuclear envelope. After Cl-4AS-1 treatment, GFP-AR showed strong directionality parallel to the nuclear envelope. In contrast, the simultaneously acquired NLS-RFP signal showed lower level of correlation in both before and after agonist treatment cells due to its fast diffusive nature. Note that the nuclear envelope boundary and the intensity differences are not the sole reasons for the observed confined anisotropic movement, as both NLS-RFP and GFP-AR showed distinct cytoplasmic/nucleus distribution.

In ligand-stimulated cells, while at the cellular scale it is clear that there is directional AR translocation from the cytoplasm to the nucleus, the much faster dynamics of nuclear transport (the reported transit time is between 1–30 ms range)^38^ requires a shorter acquisition interval to detect than our 50 ms/frame. However, we observed that GFP-AR showed strong anisotropic correlation along the nuclear membrane compared to before ligand treatment (Fig. 4). To rule out that this observation was solely the result of nuclear motion, we simultaneously acquired signals from NLS-RFP, which contains the nuclear transport sequence and localizes in the nucleus but does not have binding domains. In both untreated and ligand-treated cells, NLS-RFP did not show the same prominent confined directional motion we observed for GFP-AR.

**Figure 5:**
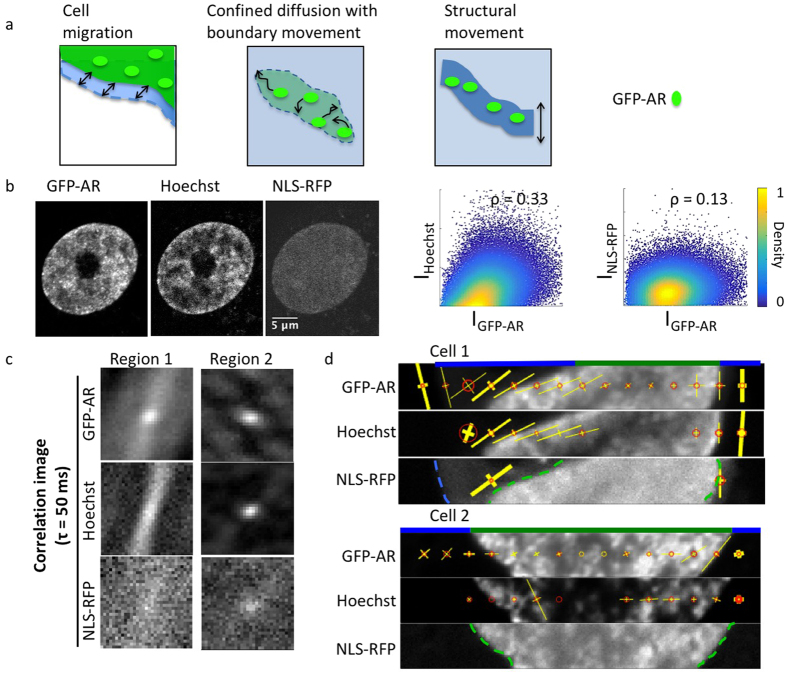
Comparison of the anisotropic motion from GFP-AR, Hoechst, and NLS-RFP after Cl-4AS-1 treatment. (**a**) The anisotropic correlation detected by STICS could be due to cell migration, confined diffusion, or structural movement. See Supplementary Figure 2 for simulations of each scenario. (**b**) With the presence of agonistic ligand, GFP-AR binds to certain locations on the chromosome, which shows more similarity to Hoechst stain (Pearson’s coefficient = 0.33) than NLS-RFP (Pearson’s coefficient = 0.13) that diffuses in the nucleus. (**c**) Two examples of GFP-AR, Hoechst, and NLS-RFP correlation from images acquired simultaneously. With the presence of ligand, GFP-AR and Hoechst exhibit similar, yet different correlation pattern in the nucleus. NLS-RFP, on the other hand, showed very weak correlation due to its fast diffusion. (**d**) Two examples of mICS analysis across a cell showing higher dynamical similarities between GFP-AR and Hoechst stain.

To test whether the GFP-AR anisotropic motion in the nucleus after agonist treatment could be explained by its binding to a structure (DNA) that moves at a time scale detectable by mICS, we simultaneously acquired signals from GFP-AR, Hoechst (to indicate DNA location), and NLS-RFP (Fig. 5). Chromatin motion has been published by several groups that tracked either DNA^39^ or nucleosomes^40^,^41^. The reported motions have scales of 10–100 ms and 100 nm, which lie within those detectable by our method (50 ms/frame, 100 nm/pixel). A clear demonstration of DNA binding as the origin of this effect would employ a version of AR that does not bind to DNA as a negative control. Such an AR variant, however, was shown to aggregate in the cytoplasm^42^ and is thus likely not appropriate for experimentation. We instead chose to use Hoechst and NLS-RFP as the positive and the negative controls, respectively. Both GFP-AR and Hoechst showed punctate patterns in the nucleus and were absent in the nucleolus, whereas NLS-RFP was uniformly distributed throughout the entire nucleus (Fig. 5b). The mICS correlation images of Hoechst and GFP-AR showed stronger isotropic correlation compared to NLS-RFP, suggesting the existence of a bound component (Fig. 5c). The anisotropic component of Hoechst and GFP-AR, although not identical, also shared correlation image shape similarities that were not found in NLS-RFP. Since overall nuclear movement would result in the same anisotropic pattern in all three fluorescent channels, and the confined diffusion model would also affect NLS-RFP, this observation suggests the anisotropic component of GFP-AR was at least partially due to the structural movement of DNA to which it bound. Figure 5d depicts two examples of mICS analysis of GFP-AR, Hoechst, and NLS-RFP across the nucleus, with overall higher similarity between GFP-AR and Hoechst.

### Simulating AR translocation with multimodal kinetics

**Figure 6:**
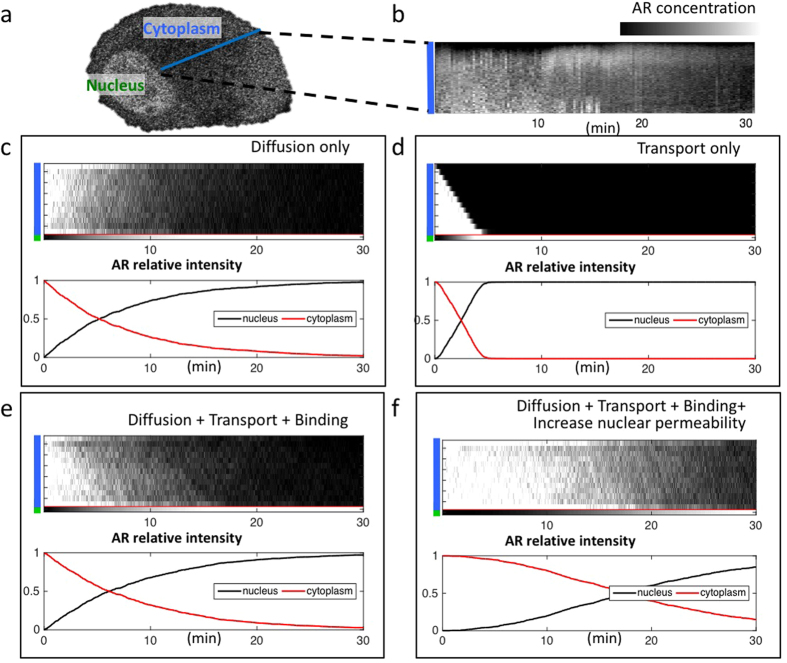
Simulating AR translocation with multimodal kinetics. (**a**) Assuming three possible AR dynamical modes in the cytoplasm (diffusion, direct transport, and binding) based on literatures and our observations, we performed Monte-Carlo simulation of AR cytoplasm to nucleus translocation, specifically focusing on AR cytoplasm distribution over time and cytoplasm-nucleus concentration change. (**b**) A kymograph extracted from the same dataset as Fig. 1a, showing the AR distribution change in the cytoplasm after adding the agonist. From (**c**,**e**), we assigned a constant probability (nuclear permeability) of AR to be transported into the nucleus when it reaches the location of nuclear envelope. (**c,d**) Both diffusion only and direct transport only model showed the ability for AR to be transported to the nucleus within 30 minutes. Noticeably, with pure diffusion, the AR cytoplasm distribution is more close to the uniform distribution that was also observed in the actual measurement (see Fig. 1a,b,e), whereas in the case of pure transport, AR formed a sharp gradient in the cytoplasm, with the highest intensity happens at the nuclear envelope, and the space close to the cell membrane showed the absence of AR. (**e**) The mixture of diffusion, direct transport, and binding at different proportions alters the average time for AR translocation. However, the slope of change was preserved, in which a faster decline of AR cytoplasm concentration happened at the beginning. (**f**) Compared to the actual measurement shown in Fig. 1c,d, a model that considers linear increase of nuclear permeability represents more closely the observation, in which AR cytoplasm portion showed a slower decrease. See Method *Simulation: AR multimodal translocation* for simulation details and Supplementary Table 1 for the parameters used.

Based on our observation of diffusive AR, AR binding, as well as previously reported AR microtubule transport^8^ in the cytoplasm, we performed 1D Monte Carlo simulations of AR translocation, specifically focusing on AR distribution in the cytoplasm, considering these three modes of motion (Fig. 6a). In this simplified model, we did not intend to capture all possible AR molecular events (such as ligand binding and dimerization), but instead to focus on properties of its motion. Each simulation began with a spatially uniform cytoplasmic AR distribution, as we observed in PC3 cells prior to ligand stimulation. To model translocation at the nuclear envelope we introduced a nuclear permeability factor, which is the probability per time step of a cytoplasmic AR molecule crossing into the nucleus when located at the envelope. The simulated nucleus acts as a one-way AR sink. During simulations we recorded changes in AR’s spatial cytoplasmic distribution and its relative proportion in the cytoplasmic and nuclear compartments. We varied the simulation parameters to assess the effects of AR transport modes and nuclear permeability on AR kinetics. The parameters we used for simulation are listed in Supplementary Table 1.

To compare with simulation results, we generated a kymograph from the nuclear envelope to the membrane using the same dataset shown in Fig. 1a. We observed no obvious cytoplasmic GFP-AR intensity gradient towards the nucleus during AR translocation (Fig. 6b), consistent with previous reports^33^,^8^. Our simulations showed that a diffusive motion component is required to maintain this spatial homogeneity. Conversely, with only active transport, AR results in a sharp cytoplasmic concentration gradient that increases over time (Fig. 6d), which is not observed in cells. This comparison is consistent with our RICS measurements, in which diffusive AR motion persisted throughout ligand-induced translocation (Fig. 1g). Furthermore, simulations revealed that either cytoplasmic diffusion or active transport alone can give rise to nuclear AR translocation (Fig. 6c,d).

Observing cytoplasmic and nuclear intensity dynamics revealed a discrepancy between experiment (Fig. 1c,d) and simulations under constant nuclear permeability (Fig. 6c–e). With nuclear permeability fixed, all combinations of active transport, binding and diffusion in the cytoplasm led to a temporal concavity having rapid depletion of cytoplasmic AR at early time points. In contrast, *in vitro* experiments resulted in a relatively delayed AR concentration change. We then simulated a varying nuclear permeability that increased linearly with time (Fig. 6f), and found the resulting concavity to be more comparable to experiment. These results imply that nuclear permeability to AR may not be constant, and instead changes gradually following exposure to agonist ligand. This delayed permeability could be attributed to a multi-step biological process, such as AR-ligand binding followed by conformational change and dissociation/association with different binding partners to facilitate nuclear transportation.

## Discussion

Dissecting AR kinetics imposes the challenges of mixed modes of motion, high spatial heterogeneity (cytoplasm versus nucleus) and the distinct response of agonist ligand binding. In the present study, we implemented different imaging techniques at several spatiotemporal scales to assess AR intracellular motion, and integrated them with simulations to provide a dynamical picture of AR translocation at the molecular scale. We detected the existence of both diffusing and binding components of AR, as well as the increased binding and confined motion in the presence of agonist ligand. We estimated that RICS and mICS capture roughly 65–75% of AR population, whereas the rest of the population is relatively immobile within the measurement time frame (~1 minute) (see Supplementary Fig. 4).

In this article we highlighted the existence of multimodal AR kinetics in prostate cancer using a model system. The PC3 cells we used lack endogenous AR and originate from metastatic neuroendocrine cancer. Thus there are caveats in generalizing these results to clinical disease. Importantly, using additional cell lines that correspond to different stages of prostate cancer (e.g. localized or androgen sensitive disease) is warranted. Adding to the complexity, even the widely used AR + prostate cancer cell line LNCaP may have multiple AR genetic variants, and its androgen responsiveness is sensitive to passage number and culture conditions^43^. We measured the kinetics of GFP-labeled rather than endogenous AR. While it has been shown that GFP-AR conserves the essential functional characteristics of AR, such as the binding affinity of agonist and transcriptional activation^3^, the kinetics may in fact differ between GFP-AR and AR.

In addition to the conventional confocal imaging, the ICS methods demonstrated in this work can be applied to study a broad array of biological systems with minimum perturbation and routine fluorescent sample preparations. The technique is particularly useful when the labeled particles under study are too dim or too dense to be individually tracked, and can capture motions of different natures. The mICS analysis we developed not only allows us to retrieve the isotropic component more accurately, but we can also decipher the biophysical origins of the anisotropic component. Using a confocal microscopy system, the spatiotemporal resolution that can be achieved with mICS is determined by two major factors: (1) the fluorophore brightness and photon collection efficiency that dictate the pixel dwell time, which limits the speed of acquisition, and (2) spatial coverage and desired pixel resolution, which dictate the number of pixels that need to be acquired. Going forward, implementing camera-based fast acquisition and multi-color acquisition can potentially offer a more detailed view of the interaction of AR with different cellular components, with higher spatiotemporal resolution.

Using multiple fluorescence microscopy assays, Van Royen *et al.*^4^ identified two types of AR–DNA binding in the nucleus: very brief interactions, and hormone-induced longer-lasting interactions, with a characteristic binding time of several seconds, comparable to our mICS results. Also similar to our observations, Brazda *et al.* reported both fast and slow components (D = 1.8–6.0 μm^2^/s and D = 0.05–0.10 μm^2^/s, respectively) of the retinoic acid receptor (RAR), a structurally similar nuclear receptor, in the nucleus^17^. They found that the RAR–agonist treatment, while shifting the RAR population towards the slower component, did not significantly change the mobilities of either the fast or slow component^17^. This is consistent with our RICS data, where we showed the diffusion coefficient was not significantly affected by the presence of ligand.

However, we observed neither evidence of dominant AR-microtubule binding nor significant direct transport in the cytoplasm, despite previous reports of AR association with dynein and increased AR-dynein interaction upon ligand induced AR nuclear translocation^8^. This difference can potentially be explained by: (1) The proportion of AR that undergoes active transport may be small compared to the binding and diffusive components; (2) The microtubule network may not be uniformly aligned (as shown in Fig. 1e), resulting in the cancellation of directionality when the analysis window is relatively large (3.2 μm by 3.2 μm) compared to a single microtubule; and (3) discrepancies between live cell experiments and fixed sample/biochemical approaches. Further experiments with higher spatiotemporal resolution may reveal more details of AR’s association with microtubule transport.

Although both treating with dynein inhibitor and taxanes showed some degree of AR translocation blockade (see Supplementary Fig. 5, also Darshan *et al.*^8^), our results suggest that this blockade may involve other mechanisms besides their effect on AR microtubule transport. In those cells that did not show translocation under dynein inhibitor or paclitaxel treatment, the intensity of cytoplasmic GFP-AR remained spatially homogeneous, without signs of lowered AR concentration at nuclear envelope proximity. This further suggests that other mechanisms besides dynein transport contributed to the lack of translocation. As illustrated in Fig. 6c, diffusion alone, albeit non-directional, may be sufficient to conduct AR translocation given a positive nuclear permeability. To change the equilibrium of AR translocation with ubiquitous AR diffusion in the cytoplasm, changes in nuclear permeability are necessary. Several studies have linked PCa with changes of nucleus related proteins^44^,^45^,^46^, and new PCa drugs targeting nuclear translocation inhibition are under developement^47^. The detailed understanding of this pathway critical for PCa growth and progression will help yield novel and needed new therapeutic approaches.

## Materials and Methods

### Cell culture and reagents

PC3, HeLa, and LNCaP cell lines were obtained from ATCC. All were maintained in RPMI1640 (Corning™, cat. no. 10–040) supplemented with 10% heat-inactivated GemCell^TM^ bovine serum (Gemini Bio-Products, cat. no. 100–500) and Penicillin- Streptomycin (Gemini Bio-Products, cat. no. 400–109). Cells were maintained at 37 °C in a humidified incubator with 5% carbon dioxide. Prior to imaging, cells were seeded on 35 mm glass bottom dish coated with poly-D-lysine (MatTek). Cells were switched to Phenol red free RPMI (Biochrom, cat.no.F1275) media supplemented with charcoal:dextran stripped FBS(Gemini Bio Products, cat.no.100-119) and L-Glutamine(Gemini Bio products, cat.no. 400-106) the night before imaging.

To produce the PC3 GFP-AR stable cell line, PC3 cells were transfected with the pEGFP-C1-AR plasmid (a gift from Dr. Michael Mancini, Addgene plasmid (#28235))^48^ using FuGENE HD Transfection reagent (Promega). Selective conditions were applied 48 hours later using 300 μg/ml G418 (Gemini, cat. no. 400–113) and media was changed every 48 hours for 2 weeks, when stable clones were visibly expanding. EGFP positive cells were selected for by fluorescence-activated cell sorting (FACS) to obtain a GFP-AR expressing cell population. The cells stably expressing GFP-AR were maintained with 200 μg/ml G418 for positive selection. The PC3 GFP cell line was generated using MISSION^®^ pLKO.1-puro-CMV-TurboGFP^™^ Positive Control Transduction Particles (*SHC003V*, Sigma-Aldrich) to transduce cells, followed by FACS. GFP-AR transient transfections of HeLa cells were performed using pEGFP-C1-AR plasmid and FuGENE one day before experiments.

Live cell microtubule labeling was done by adding 100 nM SiR-tubulin (Spirochrome) to the dish and incubating overnight. NLS-RFP transfection was done by adding 15 μl CellLight Nucleus-tagRFP BacMam 2.0 (Life Technologies) to the dish and incubating overnight. Hoechst 33342 (Thermo Fisher) was added 30 minutes prior to imaging at final concentration of 1 μg/ml. The synthetic AR agonist Cl-4AS-1 (Tocris) was applied to cells at final concentration of 20 nM; Ciliobrevin A (Tocris) was applied at final concentration of 10 nM; Paclitaxel (Thermo Fisher, cat. no. P3456) was applied at final concentration of 1 μM.

### Live cell imaging

Cells were maintained on the microscope stage in an environmental chamber with controlled temperature, CO_2_ level, and humidity throughout the experiment. Images were obtained using 63 × 1.15 NA water immersion objective mounted on Zeiss LSM-780 inverted confocal microscope (Carl Zeiss). Hoechst signal was excited at 405 nm and collected at 407–485 nm; GFP signal was excited at 488 nm and collected at 495–557 nm; NLS-tagRFP signal was excited at561 nm and collected at 567–735 nm; SiR-tubulin signal was excited at 633 nm and collected at 637–753 nm.

Depending on the spatial and temporal resolution required, acquisition parameters were adjusted accordingly: For cellular level translocation, 512 × 512 pixels were acquired for over 100 frames at the speed of 15 to 20 s/frame. For RICS data acquisition, 128 × 128 pixels were acquired for 100 to 200 frames with pixel size of 80 nm and pixel dwell time of 6.3 μs; images were acquired continuously. For mICS data acquisition, 256 × 32 pixels were acquired for 1000 frames with pixel size of 100 nm and pixel dwell time of 1.27 μs; images were acquired continuously with frame rate of 49.6 ms. For comparing the effect of Ciliobrevin A and paclitaxel, 3 × 3 tile scans of 512 × 512 pixels (415 nm/pixel) were acquired.

For FRAP experiment, a total of 30 pre-bleach images were acquired (592 ms/frame, 256 × 256 pixels, 177 nm/pixel), followed by a single square photobleach (100% ATOF). 80 frames were acquired immediately after photobleaching. The immobile fraction was determined as described in Day and Schaufele^49^.

### Western blot

Cell lysates containing 20 μg of protein were separated by 10% SDS–PAGE (Bio Rad, cat. no. 4568034) and transferred to PVDF membranes (Bio Rad, cat. no. 1704156). The blots were probed with polyclonal antibodies against AR (H-280) (Santa Cruz Biotechnology, sc-13062). Horseradish peroxidase-linked sheep antibody against rabbit IgG (GE Healthcare, cat. no. NA934V) and mouse IgG (GE Healthcare, cat. no. NXA931) were used as secondary antibodies. The signal was detected using enhanced chemiluminescence (Thermo Scientific, cat. no. 32106). Quantifications were performed using ImageJ 1.46r.

### Luciferase reporter gene assays

Reporter gene assays were accomplished with the constructs pGL4.72 ARE2 TATA, kindly provided by Andrea Koehler and Helmut Klocker^50^, using the Luciferase Reporter Assay System (Promega, cat. no. E1910) according to the manufacturer’s instructions. Cells were transfected with 100 ng DNA/well in 96-well format and kept in phenol-free medium with 10% charcoal stripped FBS (Gemini Bio-Products, cat.no. 100-119) for 24 hours post transfection and then treated with 20 nM Cl-4AS-1 for additional 24 hours before luciferase activity was measured in a GloMax^®^ 96 Microplate Luminometer (Promega).

### Data analysis: Co-localization

For each value of pixel shift (x), Pearson’s correlation coefficient (r_p_) was calculated according to Steensel *et al.*^51^


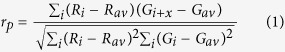


where R_i_ and G_i_ are the intensity values of pixel i of channel R and channel G, respectively. R_av_ and G_av_ are the average values of R_i_ and G_i_, respectively. We performed pixel shifts from −4 to 4 in both the x and y directions.

### Data analysis: RICS

The detailed steps for performing RICS data acquisition and analysis was performed as previously described^23^ using the simFCS package (The Laboratory for Fluorescence Dynamics, University of California, Irvine; available at http://www.lfd.uci.edu). In brief, we first temporally high-pass filtered the images by subtracting a moving average of 10 frames from the time lapse images to remove slow motion that may be due to cell movement. Then we computed the intensity-normalized spatial correlation within each frame using:


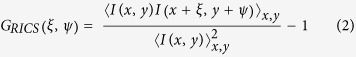


Here *I* is the intensity, ξ and ψ are the spatial increments in the x and y directions respectively, and the angle bracket denotes an average over all pixels. We averaged these image correlations across all frames and then fit this average to a 2D diffusion model to retrieve the diffusion coefficient. The RICS model is formulated as:


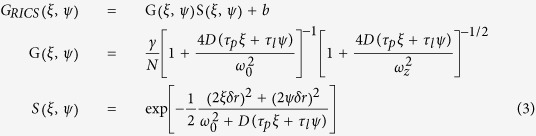


Here G_RICS_(ξ, ψ) is broken up into two parts: G(ξ, ψ), which is related to molecular dynamics, and S(ξ, ψ), which is related to scanning optics, plus the background term *b. N* is the number of particles in the focal volume; γ depends on the shape of the illumination volume; *D* the diffusion coefficient; τ_p_ the pixel dwell time; τ_l_ the time between lines; δr the pixel size; and ω_0_ the beam waist.

### Data analysis: mICS

An overview of mICS analysis is shown in Fig. 2. We first corrected the time-lapse images for bleaching using the method of Ries *et al.*^52^, which maintains constant both the image mean and its variance^52^:


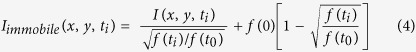


where f(t_i_) is the spatial average intensity at t = t_i_.

Second, to focus on spatial fluctuations due to motion we removed the time-averaged image using:


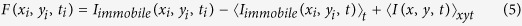


As discussed by Hebert *et al.*^24^, the spatiotemporal image correlation function is defined as


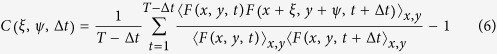


where ξ and ψ are the x and y spatial increments respectively, and the angle bracket indicates the average over all available spatial locations in both x and y directions. *T* is the total number of images in the time series. The value C represents the average cross-correlation function for all pairs of images separated by a lag time of ∆t (>0). We fitted the resulting correlation image for each lag time with a two-component (isotropic + anisotropic) Gaussian mixture model including a spatially uniform noise term ε:


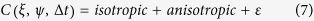


where


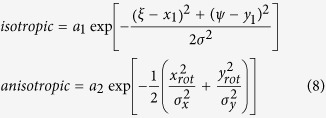


with


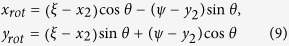


This model includes an anisotropic term that is not needed if the motion is actually isotropic. Thus we first determined whether the data are well fit by the purely isotropic model. We performed a one-sided likelihood ratio test between model fits with and without the anisotropic term included. If the likelihood ratio test p-value is less than 0.05 we included the anisotropic component in the model. Otherwise we defined the motion as isotropic.

We then assessed the maximum likelihood model’s fit and the correlation magnitude to decide whether the correlation function had a significant Gaussian component. We applied two criteria. First, we used the coefficient of determination (R^2^) as the goodness of fit. Data for model fits with R^2^ ≤ 0.1 were typically without spatial structure and we considered them to be noise 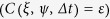. When R^2^ > 0.1 we proceeded to evaluate the amplitudes of the Gaussian components (a_1_ and a_2_). We displayed a model component (isotropic or anisotropic) only if its corresponding amplitude is greater than 10^−4^.

We performed all data analysis with custom software written in Matlab (MathWorks, version R2015a). The software is available upon request.

### Kymograph

Intensity profiles are extracted from a time-lapse image of 32.5 minutes (512 × 512 pixels, 142 time points, 1.58 us pixel dwell time, 15 seconds interval). A Matlab script uses the function *improfile* to extract the intensity profile from edge of nucleus to edge cell. Repeating the operation on different time points produces the intensity profile as a function of time.

### Simulation: AR multimodal translocation

We devised a 1-dimensional Monte-Carlo simulation for AR cytoplasm to nucleus translocation to better understand the role of various molecular kinetics and nuclear permeability.

At time 0 (in the environment of no ligand presence), we assume all AR is uniformly distributed within cytoplasm. The distance from cell membrane to nucleus was set to 50 μm. A total of 500 AR were simulated for 1800 seconds at time step equals to 1 second. We assume in the cytoplasm, at each time point AR can either choose diffusion, stand still (due to possible binding or insufficient dynein^53^), or direct transport. The diffusion follows normal distribution with mean set to 0 and standard deviation √(2D), where D is the diffusion coefficient. The direct transport also follows normal distribution but with mean equal to dynein velocity and standard deviation = (dynein velocity)/2, based on the estimation from the literature^54^,^55^.

Note that for diffusion it is possible for AR to go towards or away from the nucleus, whereas the active transport is unidirectional, only towards the nucleus (assuming dynein transport)^8^. We assigned different probabilities of AR being in these three possible modes (Table 1). When AR reaches nucleus, there is certain probability for AR to be transported into nucleus (nuclear permeability), or else AR will be retained in the cytoplasm and continue the motion. All simulations were carried out in the Matlab programming environment.

## Additional Information

**How to cite this article**: Chiu, C.-L. *et al.* Intracellular kinetics of the androgen receptor shown by multimodal Image Correlation Spectroscopy (mICS). *Sci. Rep.* **6**, 22435; doi: 10.1038/srep22435 (2016).

## Supplementary Material

Supplementary Information
