# Supplementary material for: Intracellular kinetics of the androgen receptor shown by multimodal Image Correlation Spectroscopy (mICS)
Source: Sci Rep. 2016 Mar 3;6:22435. doi: 10.1038/srep22435 (PMC4776155; doi:10.1038/srep22435)
Supplement: Supplementary Information [file srep22435-s1.pdf]

Title

Intracellular kinetics of the androgen receptor shown by multimodal Image Correlation Spectroscopy (mICS)

Authors

Chi-Li Chiu[1]#, Katherin Patsch[1]#, Francesco Cutrale[2], Anjana Soundararajan[1], David B Agus[1], Scott E Fraser[2], Daniel Ruderman[1]*

Author affiliations

1: Center for Applied Molecular Medicine, University of Southern California, USA

2: Translational Imaging Center, University of Southern California, USA

*Corresponding author: ruderman@usc.edu

#These authors contributed equally to this work

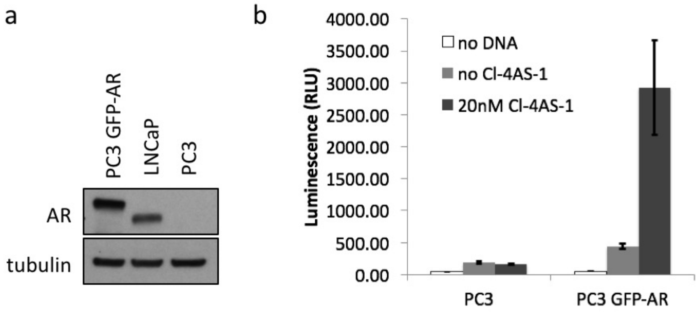

Supplementary figure S1: PC3 GFP-AR cell line physiological function assessment.
(A) Representative Western blot demonstrated levels of AR expression in the clonal PC3 GFP-AR cell line and LNCaP cell line. The PC3 GFP-AR expression level was 1.9±0.36 fold compared to LNCaP AR expression (Quantification of 3 independent cell lysates). AR protein levels were normalized to tubulin. (B) AR-dependent luciferase reporter assays were performed to assess the functionality of the GFP-AR protein upon ligand stimulation (N = 3 biological replicates for each condition, each biological replicate was performed in triplicate wells). PC3 GFP-AR cells and parental AR-negative PC3 cells were transfected with reporter vectors carrying androgen response elements (AREs) or with no reporter vectors (No DNA) as negative control. PC3 GFP-AR cells with ligand stimulation showed significantly higher luciferase activity compared to the non-stimulated group (two-sided t test, p value = 0.02), they were also higher than PC3 cells with ligand stimulation (two-sided t test, p value = 0.03).

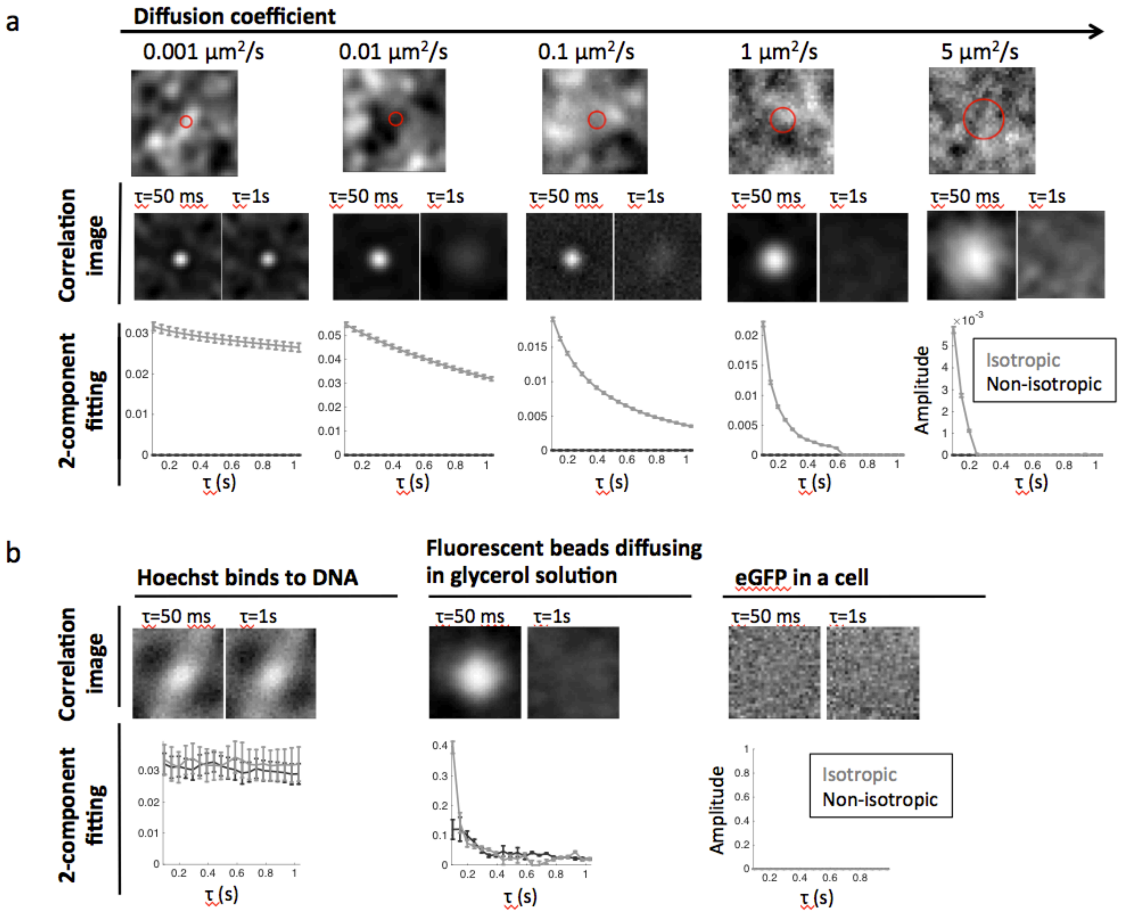

Supplementary figure S2: mICS analysis under different diffusion rate.

(A) Simulations that replicate actual image acquisition parameters (same pixel size, frame rate, and comparable point spread function (PSF)) were performed with different particle diffusion coefficients. Simulated images were then analyzed by mICS analysis. Here we show the original images overlaid with the red circles that highlight the fitting feature at τ= 50 ms, as well as the correlation images and correlation amplitude change over time delay τ.

Faster diffusion results in larger circle, and very slow diffusion (D < 0.01 $\mu m^2/s$) will have the circle close to the size of PSF convoluted with particle size. With frame rate of 50 ms, the fastest diffusion can be captured by mICS analysis is ~D = 1 $\mu m^2/s$. Faster diffusion does not generate any correlation. With diffusion only, the isotropic component dominates the correlation, and no anisotropic component was identified. (B) The high affinity nucleic acid stain Hoechst showed an example of persistent binding. Both isotropic and anisotropic components showed long correlation patterns with constant amplitude at second scale. Fluorescent beads diffusing in glycerol solution showed higher correlation at short time delay and gradually diminished to background level. The shape is consistent with the characteristic of diffusion, which has the amplitude inversely proportional to the delay time. eGFP in PC3 cell cytoplasm represents an example of fast diffusion (~30 $\mu m/s^2$). No significant correlation was captured by mICS analysis under our experimental condition of 50 ms/frame.

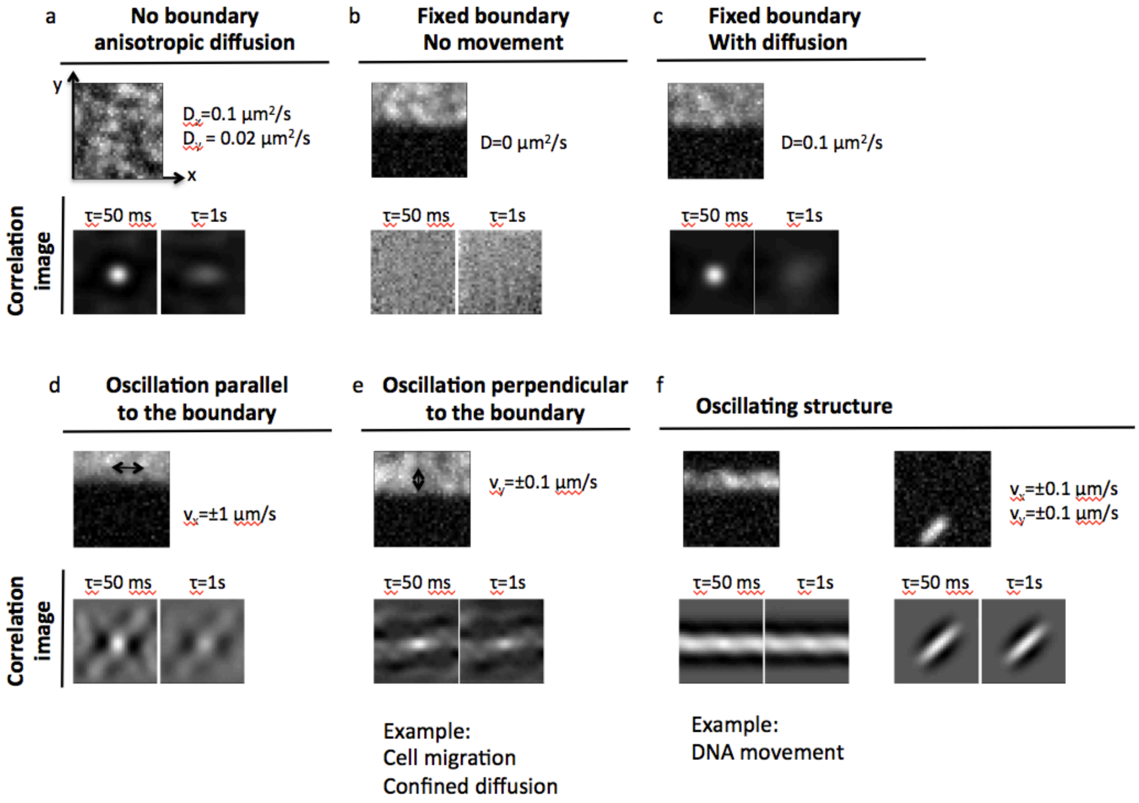

Supplementary figure S3: mICS analysis of simulated anisotropic movement.
Six scenarios were simulated to show the contribution of different motions to the anisotropic component in mICS analysis. (A) Without any boundary, anisotropic diffusion can be measured by the extent of FWHM increase for each axis. (B) With fixed boundary but no particle movement, the intensity difference was corrected by the immobile fraction removal step and resulted in no correlation. (C) Diffusion within the boundary gave the same result as diffusion without any boundary, again showing that the correlation only picks up the intensity fluctuation. (D and E) If the intensity fluctuation is parallel to the boundary, the correlation shows the magnitude of the oscillation. However, if the intensity fluctuation is perpendicular to the boundary, not only the particle vibration but also the area of boundary change will be picked up by the correlation, especially with high density fluorescent molecules. (F) With an oscillating structure, the correlation is similar to the shape of the structure.

a

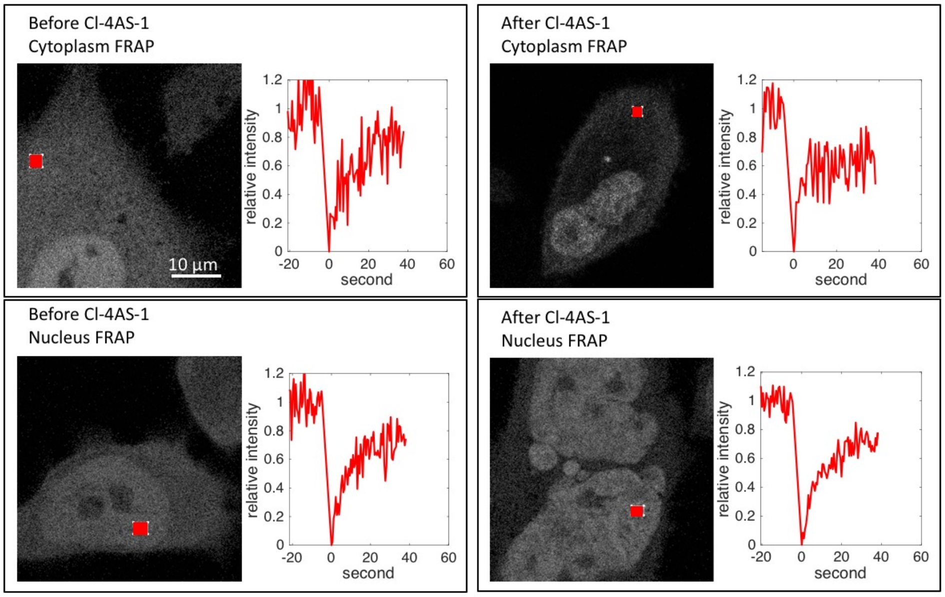

b

Immobile fraction	Before Cl-4AS-1		After Cl-4AS-1	
	nuc	cyt	nuc	cyt
Mean	0.32	0.35	0.29	0.24
Standard error	0.04	0.05	0.04	0.05
N	5	5	10	4

Supplementary figure S4: Estimate immobile fraction through FRAP.
(A) representative images showing FRAP measurement in the cytoplasm or nucleus either before Cl-4AS-1 treatment or after. Red boxes indicate the photo-bleached region. Normalized average intensity of bleached region is plotted over time, where 0 seconds is the time of bleaching. The immobile fraction was estimated by the difference between the pre-bleach intensity and the recovered intensity. (B) Summary of immobile fraction estimation. N: number of cells.

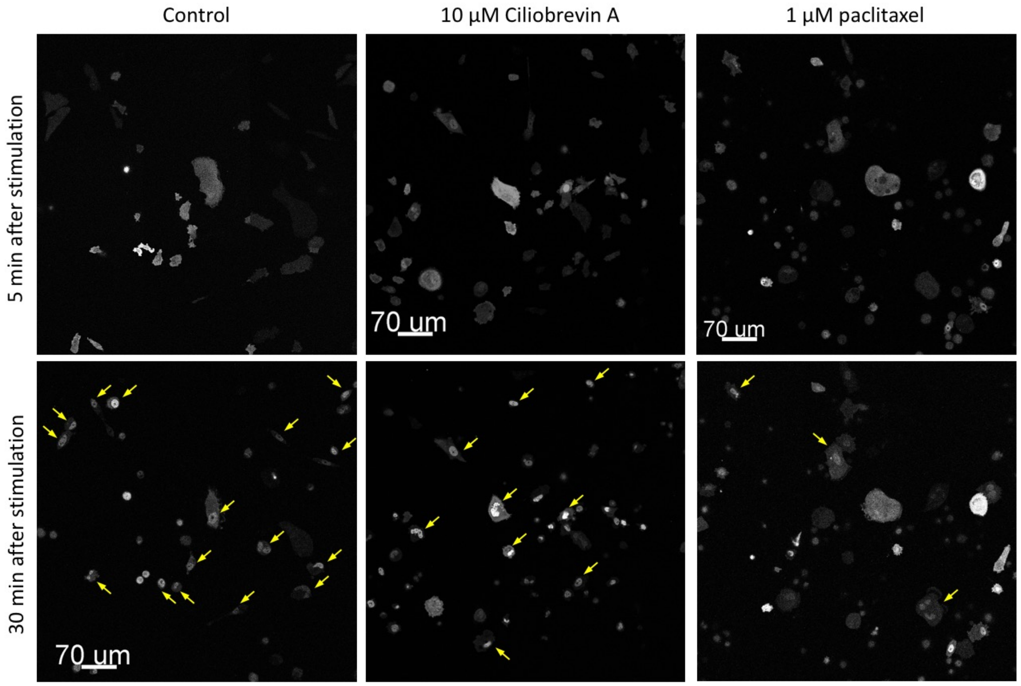

Supplementary figure S5: AR translocation under Ciliobrevin A and paclitaxel treatment. The PC3 GFP-AR cell line was stimulated with 20 nM Cl-4AS-1 either with no prior treatment (control), incubated with 10 μM Ciliobrevin A (dynein inhibitor) for 5 minutes, or incubated with 1 μM paclitaxel overnight. Cells showing clear AR translocation are indicated by arrows. While much fewer cells showed AR translocation under paclitaxel treatment, no cells showed cytoplasmic AR depletion near the nuclear envelope, either with dynein inhibitor or paclitaxel treatment. This suggests that taxane-induced AR translocation inhibition may involve other mechanisms than blocking dynein-mediated AR transportation.

Parameters		Reference
Membrane to nucleus distance	50 μm	
Time step size	1 s	
Diffusion coefficient (D)	9 $\mu m^2/s$	This paper
Direct transport velocity (T)	0.2 ± 0.1 μm/s	Reck-Peterson et al.[54], McKenney et al.[55]
Binding (B)	0 μm/s	
**Condition**	**D, T, B percentage**	**Nuclear permeability**
D only (Fig 6c)	100%, 0%, 0%	5%
T only (Fig 6d)	0%, 100%, 0%	5%
D+T+B (Fig 6e)	50%, 20%, 30%	5%
D+T+B+nuc (Fig 6f)	50%, 20%, 30%	0-5.4%, linear increase

Supplementary Table 1: AR translocation with multimodal kinetics simulation parameters.
